# The Content of Total Carotenoids, Vitamin C and Antioxidant Properties of 65 Potato Cultivars Characterised under the European Project ECOBREED

**DOI:** 10.3390/ijms241411716

**Published:** 2023-07-20

**Authors:** Beata Tatarowska, Dorota Milczarek, Jarosław Plich

**Affiliations:** Plant Breeding and Acclimatization Institute—National Research Institute in Radzików, Młochów Division, Department of Potato Genetics and Parental Lines, Platanowa Str. 19, 05-831 Młochów, Poland; d.milczarek@ihar.edu.pl (D.M.); j.plich@ihar.edu.pl (J.P.)

**Keywords:** potato, health compounds, carotenoids, vitamin C, total antioxidant activity

## Abstract

The aim of this study was to determine the effect of cultivars on the concentration of antioxidant compounds: total carotenoid content (TC) and vitamin C (VC), and their correlation with the total antioxidant activity (TAA) in 65 potato cultivars (*Solanum tuberosum*) from 10 countries. The TC content revealed a highly significant effect of the year (Y), cultivar (C) and flesh colour (FC). The TC ranged from 101.5 µg 100 g^−1^ DM (in cv. Kelly) to 715 µg 100 g^−1^ DM (in cv. Mayan Gold). The TC values were weakly correlated with years and higher in yellow-fleshed potatoes than in white-fleshed potatoes (319.9 vs. 175.6 µg 100 g^−1^ DM, respectively). The VC content ranged from 1.0 mg 100 g^−1^ FM (in cv. Bzura) to 14.8 mg 100 g^−1^ FM (in cv. Twinner). The content of VC were higher in yellow-fleshed (6.5 mg 100 g^−1^ FM) than in white-fleshed potatoes (5.8 mg 100 g^−1^ FM). The highest TAA were observed in cvs. Colleen, Basa, Triplo, Gatsby, Ditta, Twinner, Riviera, Michalina, Damaris, Belmonda, Ambo, Savinja, 12-LHI-6. For these cultivars, the FRAP values were 0.53 µmol TE 100 mg^−1^ DM and DPPH 0.55 µmol TE 100 mg^−1^ DM. The lowest TAA were observed in cvs.: Owacja, Mayan Gold, Kokra, Magnolia and Kelly. For them, the FRAP and DPPH values were slightly above 0.2 µmol TE 100 mg^−1^ DM. It was shown that the concentration of TC in potato tubers has an impact on TAA.

## 1. Introduction

The potato is the fifth most important staple food crop in the world, which supplies energy and some nutritionally relevant ingredients [1,2]. The nutritional value of potato tubers is mainly characterised by the presence of essential amino acids, high contents of starch and dietary fibre, as well as a low concentration of fats. Potato tubers also contain important levels of bioactive compounds and antioxidants, including phenolic acids, carotenoids and flavonoids. In the human body, these bioactive compounds may act as very strong antioxidants. In addition to scavenging free radicals, multiple activities of antioxidants include inactivating metal catalysts by chelation, reducing hydroperoxides to stable hydroxyl derivatives, and interacting synergistically with other reducing compounds [3,4]. Therefore, a higher consumption of potato tubers may increase the level in blood and tissues and acts against oxidative stress, which is responsible for damage to lipids, proteins, enzymes and DNA, resulting in chronic diseases such as cancer or cardiovascular disease (CVD) [5,6,7]. These compounds and their antioxidative potential, which describes potentially health-promoting properties of potato tubers, are determined by genetics as well as environmental factors. Currently, the market’s demand for food rich in antioxidant compounds is increasing. Plant breeding has invested significant resources in the selection of potato cultivars with a higher content of carotenoids, flavonoids, vitamin C and, consequently, a higher antioxidant potential [8,9,10]. One of the methods that allows us to improve the composition of these compounds is breeding focused on biofortification (increased nutritional security), which has the task of enriching the nutritional value of the product by supplementing it with bioavailable nutrients.

In this research, 65 potato cultivars from the work collection created within the European project ECOBREED were used. Cultivars which were selected for the collection were characterised by high or increased resistance to *Phytophthora infestans* and were commercially available in the European seed market. These cultivars come from 10 countries and are used to identify the most important characteristics for organic farming systems. The aim of this research was to evaluate the variability of the bioactive compounds (total carotenoids and vitamin C) in 65 potato cultivars. In this study, the total antioxidant activity of potato tubers depends on the content of individual phytocompounds which depend on the total antioxidant activity of the tubers. In addition, cultivars were screened for the presence of dominant allele 3 in the *Chy2* locus.

## 2. Results and Discussion

Potato has been identified by the Food and Agriculture Organization of the United Nations (FAO) as a basic and sustainable food for the growing world population [11]. Therefore, it is very important to understand its contribution to both our daily and long-term health. It is very important to develop cultivars with increased antioxidant capacity as ‘functional foods’ and encourage potato consumers to buy such cultivars, rich in carotenoids, vitamin C and other pro-health compounds.

Potato cultivars show great variability in terms of carotenoid accumulation in tubers. The concentration of carotenoids in potato tubers is affected by several factors such as genotype, agronomic factors, post-harvest storage, cooking and processing conditions [12,13,14,15,16].

**Figure 1:**
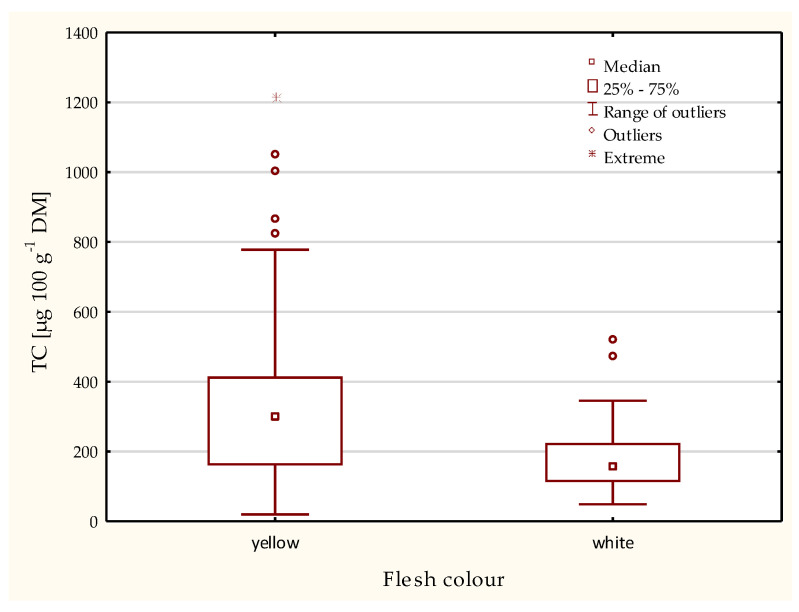
Mean values of total carotenoid content (TC) in two groups of potato with white and yellow flesh.

In our study, 65 cultivars were evaluated YI. Based on the values obtained for YI, cultivars were divided into two groups: white and yellow flesh. In total, 18 cultivars were classified as white-fleshed and 47 as yellow-fleshed. The YI of the evaluated white-fleshed potato cultivars ranged from 35.5 to 48.8 and in yellow-fleshed cultivars ranged from 50.3 to 80.4 (Table 1). The values of the YI range from 35.5 for cv Kelly to 0.4 to cv. Mayan Gold (Table 1). Also, the 65 potato cultivars differed significantly in terms of their total carotenoid content. The three-year mean TC content ranged from 101.5 µg 100 g^−1^ DM (in cv. Kelly) to 715 µg 100 g^−1^ DM (in cv. Mayan Gold) (Table 1). The examined cultivar Mayan Gold is a diploid that has *S. phureja* in its ancestry. This potato species is reported to be a good source of high total carotenoid content. The results of ANOVA showed that TC is also significantly influenced by the factor year (Y) and the correlations between the TC values between individual years were rather weak (r_2019×2020_ = 0.43*; r_2019×2021_ = 0.26*; r_2020×2021_ = 0.31*; significant at *p* < 0.05) (Table 2). Differences in rainfall intensity and distribution were probably important factors contributing to TC content in potato tubers in individual years (Figure S1). In the group with the highest TC, there were 11 cultivars: Otolia, Bionta, Anuschka, Belmonda, Carolus, Gardena, Salome, Belana, Twinner, Caprice, and Mayan Gold. The mean TC values from three years research for these cultivars range from 422.0 to 715 µg 100 g^−1^ DM (Table 1). In the group with the lowest of TC 13 cultivars were included: Kelly, Yona, Savinja, Nofy, Basa, Valor, Botond, Sarpo Mira, Riviera, Vipava, Tinca, Bzura and Erica. The mean values of these cultivars from three years of research range from 101.5 to 168.6 µg 100 g^−1^ DM (Table 1). The highest mean value of TC was recorded in 2020 (Table 1). The mean values of TC were significantly higher in potatoes with yellow-flesh (319.9 µg 100 g^−1^ DM) than in white-fleshed potatoes (175.6 µg 100 g^−1^ DM) (Table 3; Figure 1). Iwanzik et al. [17] reported a range from 27 to 74 µg 100 g^−1^ FW for TC in white-fleshed potato cultivars. In yellow-fleshed potatoes, TC is usually higher, up to 560 µg 100 g^−1^ FW [18,19,20]. In diploid clones of *Solanum* species, reported levels of TC are much higher and reached more than 2000 µg 100 g^−1^ FW [21]. A very high TC of up to 3850 µg 100 g^−1^ FW was found in cultivars from the *Andigenum* group [22]. Othman [23] evaluated 32 potato cultivars grown in New Zealand. Agria, a dark-yellow-fleshed cultivar, was found to have the highest TC (169.57 µg/g DW), the lowest TC was found in the white-fleshed cultivar Moonlight (1.18 µg/g DW). He also reported that genotype, location and their interaction were significant. Tatarowska et al. [10] reported a TC range from 5.57 to 20.20 mg kg^−1^ FW and proved that several factors, location, year, genotype, and their interactions influence on carotenoid accumulation.

Two main loci have been shown to control potato tuber flesh colour. The Yellow (Y) locus was shown to co-segregate with the *β-Carotene Hydroxylase 2* (*CHY2*) gene [24]. A dominant *Y* allele of this locus was associated with a high flesh carotenoid content and showed increased expression of the *CHY2* transcript [25,26]. The Orange (Or) locus was associated with high amounts of flesh zeaxanthin [27]. The *Or* locus was found to co-segregate with the *Zeaxanthin Epoxidase* (*ZEP*) gene. All orange genotypes carry only a recessive *zep* allele (*zep1*) characterised by the insertion of a non-LTR retrotransposon into intron 1 and by reduced steady-state levels of the *ZEP* transcript.

**Figure 2:**
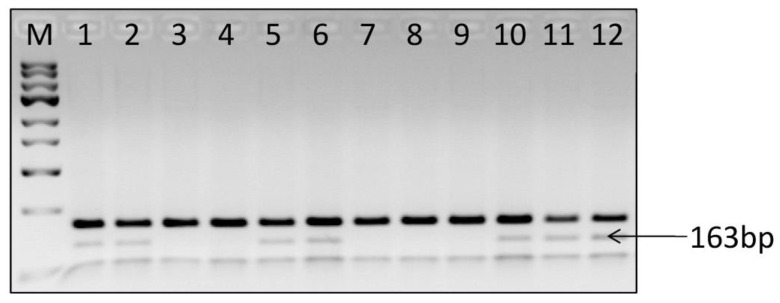
Amplification of the CAPS marker linked to the *Chy2* gene allele 3. Cultivars having *Chy2* gene allele 3 are shown in lanes 1, 2, 5, 6, 10, 11 and 12, respectively. Lanes 3, 4, 7, 8 and 9 show that cultivars lack the *Chy2* gene allele 3. Lane M contains the 100 bp DNA ladder.

Brown et al. [24] examined 21 yellow fleshed and 28 white fleshed potato cultivars and breeding clones for the presence of a unique allele of *bch* associated with yellow flesh colour and obtained the expected for gene product from all yellow-fleshed cultivars and breeding clones tested. The same primers amplified no product from any of the white-fleshed clones tested. In the research by Sulli et al. [28], the dominant *CHY2* allele 3, previously found to be a major determinant of carotenoid accumulation, was present only in yellow or orange-fleshed clones. Goo et al. [29] also observed a higher expression level of *Chy2* associated with yellow flesh colour in a small number of potato cultivars. In our investigation, the dominant *Chy2* allele 3 was amplified in 50 of the 65 cultivars tested (Table 1). In Figure 2, the amplification of CAPS marker linked to *Chy2* gene allele 3 is presented. Cultivars with amplified diagnostic fragment were both yellow and white fleshed. Cultivars without this fragment were also yellow and white fleshed. However, this fragment was absent in more than 44% of white-fleshed cultivars and only in ~15% of yellow-fleshed cultivars. The examined cultivars with the marker had higher YI and TC than the cultivars without the marker, but the differences were statistically significant in the case of YI but not TC (Table 4). In conclusion, the examined marker of *β-Carotene Hydroxylase 2* can help in selection of potato clones/cultivars with high carotenoid content, but its usefulness is restricted.

**Figure 3:**
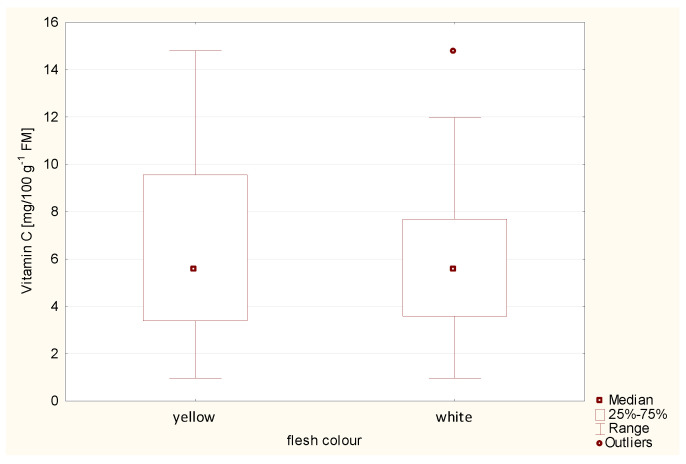
Content of vitamin C in two groups of potatoes with white and yellow flesh.

One of the powerful antioxidants in plants is vitamin C. In potato tubers, it occurs as L-ascorbic acid and dehydroascorbic acid in the acid oxidation position [30]. Potatoes are one of the most important sources of vitamin C, but its value in human nutrition is often underestimated [31]. Vitamin C is the major naturally occurring inhibitor of enzymatic browning of potatoes [32]. In the human body, it acts as an antioxidant [11] and plays an important role in the protection against oxidative stress [33]. The VC content in potato tubers influences many factors, such as cultivar, maturity, year, method and growing conditions and location [16,34,35,36]. Various studies have measured large differences between potato cultivars in their VC content from 7.54 to 46.0 mg 100 g^−1^ FM [34,37,38,39,40]. Our studies showed a great variation between potato cultivars’ VC content. The mean VC values for 65 cultivars are presented in Table 1. The average VC content in 65 cultivars ranged from 1.0 to 14.8 mg 100 g^−1^ FM. The highest level of VC was noted for cultivars: Twinner (14.8 mg 100 g^−1^ FM), 12-LHI-6 (12.9 mg 100 g^−1^ FM), Tinca (12.4 mg 100 g^−1^ FM), Levante (12.3 mg 100 g^−1^ FM), Kelly (12.0 mg 100 g^−1^ FM), Karlena (11.9 mg 100 g^−1^ FM), Agria (11.8 mg 100 g^−1^ FM) and Colleen (11.6 mg 100 g^−1^ FM) (Table 1). In Figure 3 are presented mean values of VC in two groups of flesh colour. Mean values of VC were higher in potatoes with yellow-flesh (6.5 mg 100 g^−1^ FM) than in potatoes with white-flesh (5.8 mg 100 g^−1^ FM), but this difference was not statistically significant (Figure 3; Table 3). One-way ANOVA analysis showed a significant influence of the cultivar on the content of VC in potato tubers (Table 2).

Similar to our research, Sawicka et al. [41] proved that in the formation of VC in potato tubers, the greatest impact is the genotypic variability. According to Brown [42], VC content on the level 20 mg/100 g^−1^ FM can raise up to 13% in the TAA of tubers. Several authors also confirm the influence of earliness on the VC content in potato tubers. Trawczyński et al. [43] and Hrabowska et al. [44] showed that a higher VC was noted for late cv. compared to early cv. According to Grudzińska and Mańkowski [5], the flesh colour significantly influenced the VC content in potato tubers. The authors observed significant differences in the VC content between tubers with yellow flesh and cream flesh. Hamouz et al. [35] determined that VC content was 2.9-times higher in red and purple potatoes than in potatoes with yellow and white flesh. Valcalcer et al. [16] proved that the highest VC content was in yellow flesh cv. and the lowest in cream flesh cv. By contrast, Hejtmánková et al. [45] showed that white and yellow potatoes did not differ in terms of VC content, similar to our research. The high diversity of VC in potato tubers shows that to conduct breeding towards the production of cultivars with high VC content is justified. Biofortification using breeding programs aimed at increasing the VC content in potato tubers is fully justified.

**Figure 4:**
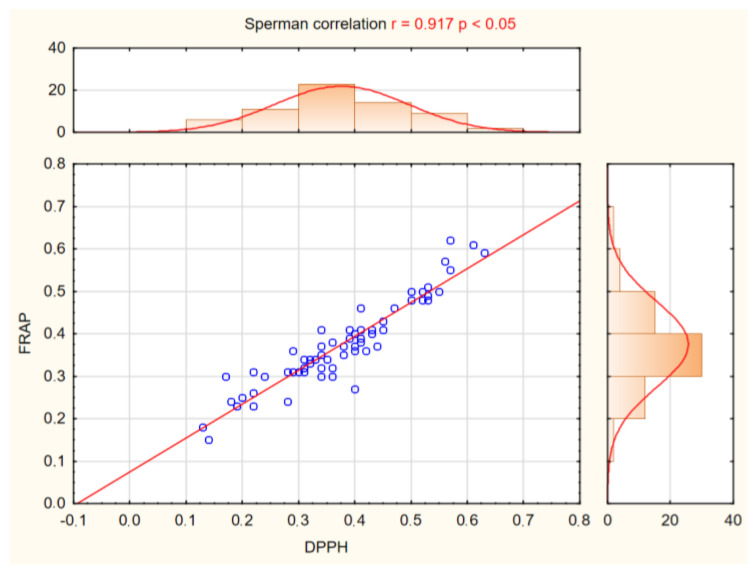
Correlation between two methods, FRAP and DPPH, used to measure TAA in tubers of 65 potato cultivars.

**Figure 5:**
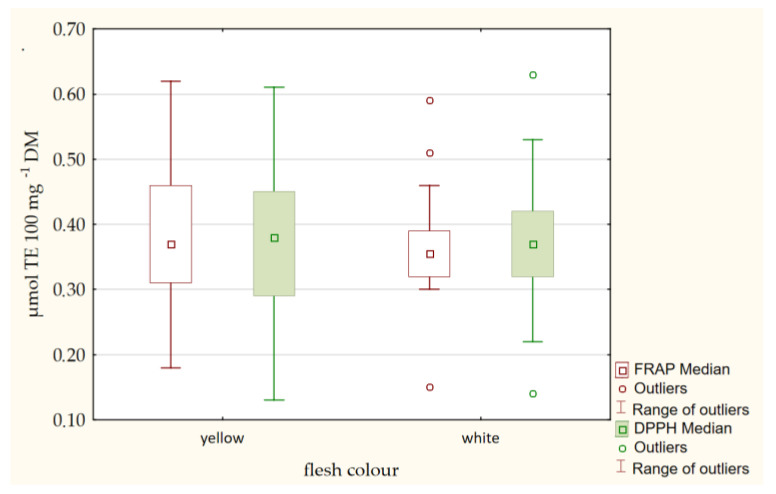
TAA of potato tubers measured by DPPH and FRAP in two groups of potato with white and yellow flesh.

The concentration of bioactive compounds in potato tubers is determined by both genetic potential and environmental factors. The purpose of these experiments is, among others, to determine the influence of genotype on the TAA of potato tubers. The antioxidant capacity of 65 potato cultivars from nine countries dedicated to organic farming was determined. Two different methods of measuring antioxidative capacity, i.e., DPPH and FRAP, were used. On the basis of these analyses conducted, it can be said that the evaluated cultivars are different in the TAA of potato tubers. The full results of the two methods and all the cultivars are presented in Table 1. For the value of FRAP and DPPH, one-way ANOVA revealed highly significant effect for cultivar (C). Higher antioxidant activity was observed in 10 cultivars: Colleen, Basa, Triplo, Gatsby, Ditta, Twinner, Riviera, Michalina, Damaris, Belmonda, Ambo, Savinja, 12-LHI-6 (Table 1). For these cultivars, the mean FRAP values were 0.53 µmol TE 100 mg^−1^ DM and the mean DPPH values were 0.55 µmol TE 100 mg^−1^ DM. Tubers of these potato cultivars have many benefits to prevent oxidative stress and potential as sources of natural antioxidant can be further used in breeding work. A lower antioxidant activity was observed in cultivars: Owacja, Mayan Gold, Kokra, Magnolia and Kelly (Table 1). For them, the FRAP and DPPH values were slightly above 0.20 µmol TE 100 mg^−1^ DM. Strong positive correlations (r = 0.917*; significant at *p* < 0.05) were found between FRAP and DPPH (Figure 4). These methods can be used interchangeably in the assessment of the TAA in potato tubers. In potato cultivars with yellow-fleshed tubers higher antioxidant activity was observed (mean DPPH = 0.39 µmol TE 100 mg^−1^ DM; mean FRAP = 0.39 µmol TE 100 mg^−1^ DM) than in white-fleshed potatoes (mean DPPH and FRAP = 0.33 µmol TE 100 mg^−1^ DM) (Figure 5), but these differences were not statistically significant (Table 3).

In the literature, can be find research showing a large variation of TAA among potato cultivars/clones, and many other factors having an impact on their level in potato tubers. For example, Wichrowska [46] reported a significantly differentiated TAA of potato tubers cv Satina measured using the FRAP method. The highest TAA was characteristic of tubers from the plots fertilized with manure, a full dose of mineral fertilization and bio-fertiliser. FRAP values ranged from 6.98 to 9.91 mmol Fe^2+^ kg^−1^ DM. Hu et al. [47] reported a significant effect of cultivar and year on TAA. In the FRAP assay, antioxidant activity ranged from 6 to 64 µmol AAE g^−1^ DW and in ORAC ranged from 42 to 168 µmol TE g^−1^ DW. Lee et al. [48] reported that the TAA was significantly higher in purple-coloured potato cultivars than in the white- and yellow-coloured potatoes. In their research, they showed that cvs. Hongyoung and Jayoung had the highest antioxidant activity. Antioxidant effect was highly correlated with polyphenol content. Kim et al. [49] reported that the effective compounds that contribute to TAA were phenolics acids. Fidrianny et al. [50] proved that the phenolic and flavonoids compounds of potato tubers are very strong antioxidants. Seijo-Rodríguez et al. [51] stated that due to the high correlation between the phenolic content and antioxidant activity, the phenolic content in potato tubers can be used as an indicator of the antioxidant activity of a tuber. The Keutgen et al. [8] reported that antioxidative capacity measured by FRAP was correlated with chlorogenic acid content (r = 0.590**; significant at *p* < 0.01) and glutathione fractions, especially with the reduced form (GSH, r = 0.692**; significant at *p* < 0.01). Hejtmánková et al. [45] did not detect a significant correlation between content of VC and antioxidant activity (R^2^ = 0.08) in unpeeled raw potato. Grudzińska and Mańkowski [5] also indicated the lack relationship between TAA and VC in unpeeled potatoes (R^2^ = 0.17).

**Figure 6:**
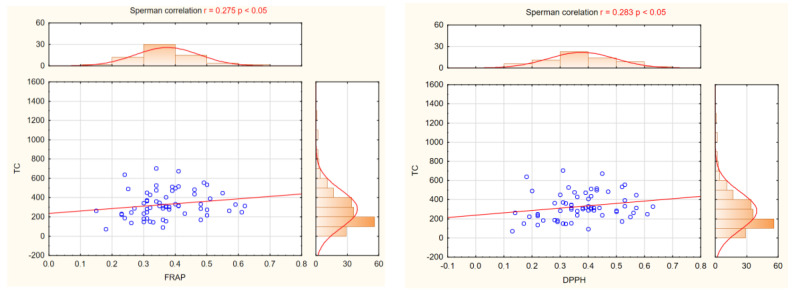
The relationship between TAA measured by FRAP and DPPH and the content of TC in tubers of 65 potato cultivars.

**Figure 7:**
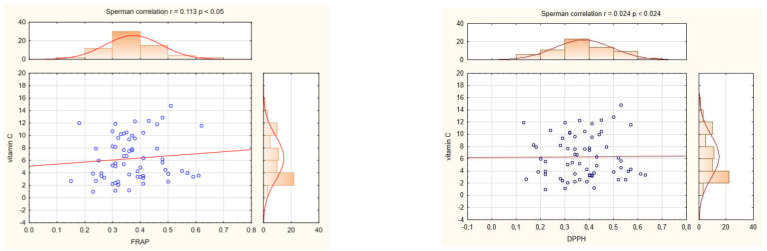
The relationship between TAA measured by FRAP and DPPH and the vitamin C content in tubers of 65 potato cultivars.

In our research, we also wanted to indicate which compounds in potato tubers have the greatest impact on TAA. The FRAP and DPPH assays indicate that TC in potato tubers contributes to their antioxidant activities. The correlation between TC and TAA measured by FRAP and DPPH was significant (r = 0.275* and r = 0.283*, respectively; significant at *p* < 0.05) (Figure 6). On the other hand, the concentration of VC in the potato tubers had no effect on TAA. The correlation between VC and TAA measured by FRAP was very low and nonsignificant (r = 0.113) and DPPH (r = 0.024) (Figure 7). Our research also reported that cultivars have significantly influence on TAA (Table 2), but not on flesh colour (Table 3).

## 3. Materials and Methods

### 3.1. Plant Material

Plant material consisted of 65 potato cultivars from 10 countries: from Poland—Bzura, Michalina, Denar, Lord, Gardena, Magnolia, Owacja, Tajfun; from Denmark—Tinca, 12-LHI-6; from Germany—Caprice, Damaris, Fidelia, Goldmarie, Karlena, Salome, Wega, Anuschka, Belana, Elfe, Otolia, Agria, Omega, Belmonda, Lilly, Granola; from Netherlands—Alouette, Carolus, Erika, Levante, Nofy, Premiere, Riviera, Twinner, Twister, Colomba, Fortus, Noblesse, Triplo, Voyager; from Austria—Bionta, Ditta; from Slovenia cvs.—Kokra, Savinja, Slavnik, Vipava; from United Kingdom—Gatsby, Casablanca, Valor, Mayan Gold, Cara, Sarpo Mira, Sarpo Shona; from Ireland—Ambo, Colleen; from Hungary—Balatoni Rozsa, Basa, Botond, White Lady—and from France—Capucine, Charlotte, Delila, Edony, Kelly, Yona. These cultivars came from the Working Collection created within the European project ECOBREED.

### 3.2. Field Experiments

All these cultivars were multiplied under field conditions. The trials were carried out for three years in the central part of Poland, in Młochów. In these experiments, a low-input system was applied (in Supplementary Materials Table S1). Pesticides were applied during the vegetation period, i.e., copper fungicides and pyrethrin (plant extract) against late blight and Novodor against Colorado beetle. All application times and dose were established in accordance with the recommendations on the labels of each plant protection product. The experimental design was a randomised complete block. In each of the two blocks (replications), the cultivars were planted in 60 hill plots. The spacing of the plants in the row was 70 cm × 40 cm. Tubers were planted at the end of April and harvested after about 130 days. The monthly average temperature and precipitation were collected from a weather station located near the experimental field. The weather conditions for the growing seasons 2019, 2020 and 2021 are shown in Supplementary Materials Figure S1.

### 3.3. Total Carotenoid Content (TC)

TC was estimated for tubers obtained from field experiments. For each potato cultivar and each repetition, three tubers were collected, cut into small cubes, frozen in liquid nitrogen, lyophilised and milled. The total carotenoid content was measured according to Milczarek and Tatarowska [52]. All analyses were performed using three technical repetitions. Analyses were conducted via three-year experiments.

### 3.4. Yellowness Index (YI)

The intensity of potato flesh colour was assessed using CR-400 Colorimeter (Minolta, Osaka, Japan). YI was calculated according to the formula:

$$YI_{E313}=\frac{100\left(C_{x}X-C_{z}Z\right)}{Y}$$

where *C_x_* and *C_z_* are illuminant and observer-specific constants; *X*, *Y*, *Z*—trichromatic values [53].

### 3.5. Genotyping of the Chy2 Loci

All tested cultivars were screened for the presence of dominant allele 3 at the *CHY2* locus. The tests were performed with the use of a DNA marker developed by Wolters et al. [26]. DNA was isolated from young leaves of all tested cultivars using a Genomic Mini AX Plant (A&A Biotechnology, Gdańsk, Poland). PCR amplification was performed with PCR Mix Plus Green, a high specificity ready-to-use master mix for PCR (A&A Biotechnology, Gdańsk, Poland), and 300 nM of each primer in a final volume of 25 μL. Standard amplification conditions were as follows: initial denaturation for 4 min at 94 °C followed by 30 cycles of 30 s denaturation at 94 °C, 30 s annealing at 55 °C and 1 min elongation at 72 °C. Reactions were completed with an elongation step of 7 min at 72 °C. The PCR with primers CHY2ex4F (5′-CCATAGACCAAGAGAAGGACC-3′) and Beta-R822 (5′-GAAAGTAAGGCACGTTGGCAAT-3′) amplified product of size 308-bp. PCR products were digested with *Alu*I restriction enzyme (Thermo Fisher Scientific, Dreieich, Germany) according to the manufacturer instructions. The CAPS markers of the *Chy2* gene were separated with a 2% ethidium bromide-stained agarose gel. In the case where the dominant *Chy2* allele 3 is present, a diagnostic fragment of 163 bp was observed, whereas all the other alleles at the same locus produce only a specific fragment of 237 bp.

### 3.6. Vitamin C (VC)

Vitamin C was estimated for tubers obtained from field experiments. For each potato cultivar and each repetition, three tubers were collected. Potato tubers were peeled and then rubbed on a plastic grater. About 6 g of sample was weighed into a 50 mL test tube. It was supplemented with an extraction solution (metaphosphoric acid, acetic acid, pH 2) at a weight of 25 g, which prevented the breakdown of vitamin C. The sample was shaken on a Thermomixer compact (Eppendorf AG, Hamburg, Germany) for 15 min at 4 °C and after this was spun for 10 min at 4 °C and 604× *g* in Centrifuge 5417R (Eppendorf AG, Hamburg, Germany). After centrifugation, the clear solution was filtered through a 0.40 µm PTFE syringe filter. The injection volume was 2 µL. The mobile phase was water: acetonitrile in relative 2:98, flow 1.5 mL min^−1^. Analyses were performed using Column Luna 3u HILIC 150 × 4.6 mm (Phenomenex, Torrance, CA, USA). Vitamin C concentrations were evaluated using ultra-high efficiency HPLC liquid chromatography kit (LC-20AD, DGU-20A 3, SIL-20AC HT, CTO-10AS) with a UV detector (SPD-20A) from Shimadzu (Kyoto, Japan).

### 3.7. Total Antioxidant Activity (TAA)

#### 3.7.1. Preparation of Samples for Analysis

The antioxidant potential for tubers obtained from field experiments was evaluated. For each potato cultivar and each repetition, three tubers were collected. They had been cut into small cubes and frozen in liquid nitrogen, lyophilised and milled. Samples (0.5 g) were extracted using ethanol (80%) and placed in an ultrasonic bath for 20 min. The extracts were transferred to falcon tubes (per 15 mL) and centrifuged at a speed of approximately 537× *g* for 10 min (centrifuge Heroeus multifuge 3 S-R, Thermo Fisher Scientific, Cleveland, OH, USA).

#### 3.7.2. Measurements DPPH

Measurements were made using the spectrophotometric method. Up to 1900 μL of DPPH radical solution in methanol with a concentration of 0.05 mg ml^−1^ with 100 μL of sample added. The mixture was prepared in this way and the end reaction was set aside for 30 min. Then, the absorbance was measured at the length of λ = 515 nm. The measurements were repeated 3 times for each sample. The results are calculated based on the calibration curve for Trolox (6-hydroxy-2,5,7,8-tetramethylchroman-2-carboxylic acid).

#### 3.7.3. Measurements FRAP

The measurements were made spectrophotometrically with a UV-Vis HITACHI U-1900 spectrophotometer (Tokyo, Japan). The absorbance was measured at 593 nm whereby 0.05 mL of the sample solution were added to 1 mL of FRAP reagent. The mixture prepared this way was incubated for 4 min at 37 °C. The FRAP results were calculated from the calibration curve for the Fe (II) standard solution. The FRAP unit was assumed to determine the ability to reduce 1 mole of iron (III) to iron (II). The results were calculated for the standard substance (Trolox) and presented as (μmol TE 100 mg^−1^ DM).

### 3.8. Statistical Analysis

The mean values for TC, VC and TAA were compared by using one- and two-way ANOVA to assess the effects of cultivar, year, and their interactions, and Tukey’s multiple range test was used to identify significant differences between means. To compare mean values for groups of clones with or without markers and white and yellow fleshed, Student’s *t*-test with unequal variances was applied. The Pearson correlation coefficient was used to estimate the relationships between the study variables. All statistical analyses were performed with the Statistica software (Dell, Round Rock, TX, USA), version 13.

## 4. Conclusions

Our results permit optimisation of the vitamin C and carotenoid content of the diet by using potato cvs., such as Twinner, Tinca, Levante, Kelly, Karlena, Agria and Colleen with high-vitamin C, and by using potato cvs., such as Otolia, Bionta, Anuschka, Belmonda, Carolus, Gardena, Salome, Belana, Twinner, Caprice, and Mayan Gold with high carotenoids content. Obtained results showed how breeding efforts have influenced the biofortification of these important phytonutrients in the potato tubers cultivars registered in European markets.

## Figures and Tables

**Table 1:** Mean values of yellow index (YI), total carotenoid content (TC), presence loci of marker *Chy2*, vitamin C (VC) and total antioxidant capacity (TAA) for 65 potato cultivars.

Cultivar	YI ^(1)^	FC ^(2)^	TC in µg 100 g^−1^ DM ^(3)^			Mean Values TC Years 2019–2021	*Chy2* Loci ^(4)^	VC ^(5)^ mg 100 g^−1^ FM 2020	TAA DPPH ^(6)^	TAA FRAP ^(7)^
			2019	2020	2021				µmol TE 100 mg ^−1^ DM 2020	
Ambo	40.8	w	119.3	332.7	166.0	206.0 AB	0	5.7	0.53	0.48
Balatoni Rozsa	47.2	w	272.1	188.8	186.0	215.6 AB	1	2.8	0.28	0.24
Botond	46.3	w	91.8	237.3	93.0	140.7 B	1	5.2	0.36	0.30
Bzura	48.8	w	211.5	232.5	52.0	165.3 AB	0	1.0	0.22	0.23
Cara	42.4	w	130.6	517.0	80.0	242.5 AB	1	6.4	0.43	0.41
Colleen	48.4	w	143.5	316.5	80.0	180.0 AB	1	11.5	0.57	0.62
Denar	44.3	w	269.7	290.7	180.0	246.8 AB	1	1.2	0.42	0.36
Gatsby	45.2	w	156.5	264.8	207.0	209.4 AB	0	4.0	0.56	0.57
Kelly	35.5	w	103.1	72.4	129.0	101.5 B	0	12.0	0.13	0.18
Kokra	45.0	w	470.1	150.0	52.0	224.1 AB	0	8.3	0.17	0.30
Owacja	47.1	w	137.1	224.4	173.0	178.2 AB	1	3.9	0.19	0.23
Premiere	46.9	w	172.6	250.3	185.0	202.6 AB	1	5.6	0.22	0.31
Sarpo Mira	47.0	w	237.3	138.7	52.0	142.7 B	1	3.9	0.22	0.26
Savinja	42.9	w	67.6	172.6	127.0	122.4 B	1	6.2	0.52	0.48
Valor	39.8	w	141.9	146.8	113.0	133.9 B	0	10.3	0.32	0.33
Vipava	35.8	w	345.6	91.8	49.0	162.2 AB	0	7.5	0.40	0.36
White Lady	45.8	w	219.5	151.6	140.0	170.4 AB	0	2.1	0.31	0.32
Yona	44.7	w	141.9	156.5	49.0	115.8 B	1	7.6	0.34	0.37
12-LHI-6	57.8	y	428.1	287.4	87.0	267.5 AB	1	12.9	0.5	0.48
Agria	65.7	y	436.2	437.8	145.0	339.7 AB	1	11.8	0.41	0.46
Alouette	59.4	y	156.5	373.1	415.0	314.9 AB	1	5.1	0.31	0.31
Anuschka	71.4	y	339.2	673.9	312.0	441.7 AB	1	10.5	0.45	0.41
Basa	55.8	y	84.5	248.6	60.0	131.1 B	1	3.6	0.61	0.61
Belana	70.4	y	405.5	497.6	821.0	574.7 AB	0	4.9	0.43	0.40
Belmonda	70.0	y	303.6	555.8	508.0	455.8 AB	1	4.5	0.53	0.49
Bionta	66.3	y	444.3	332.7	517.0	431.3 AB	1	3.4	0.40	0.40
Caprice	65.3	y	505.7	512.2	1000.6	672.8 AB	1	3.4	0.41	0.39
Capucine	68.3	y	390.9	492.8	64.0	315.9 AB	1	6.0	0.20	0.25
Carolus	58.6	y	358.6	314.9	863.0	512.2 AB	1	10.5	0.38	0.35
Casablanca	50.3	y	114.4	169.4	290.0	191.3 AB	1	9.4	0.29	0.36
Charlotte	58.5	y	151.6	313.3	249.0	238.0 AB	1	3.5	0.34	0.41
Colomba	51.3	y	373.1	319.8	93.0	262.0 AB	1	2.3	0.39	0.41
Damaris	53.5	y	298.8	276.1	112.0	229.0 AB	1	3.9	0.50	0.50
Delila	52.3	y	348.9	185.6	133.0	222.5 AB	1	2.4	0.29	0.31
Ditta	62.6	y	184.0	449.1	225.0	286.0 AB	1	4.3	0.57	0.55
Edony	61.2	y	127.4	303.6	88.0	173.0 AB	1	9.6	0.41	0.38
Elfe	68.8	y	184.8	483.1	328.0	332.0 AB	1	7.9	0.47	0.46
Erika	59.0	y	130.6	318.2	57.0	168.6 AB	1	3.2	0.41	0.41
Fidelia	65.1	y	281.0	347.3	161.0	263.1 AB	1	6.8	0.34	0.35
Fortus	59.0	y	295.5	305.2	120.0	240.2 AB	1	7.6	0.38	0.37
Gardena	59.3	y	212.3	316.5	1049.0	525.9 AB	1	3.8	0.42	0.36
Goldmarie	64.5	y	307.7	473.4	129.0	303.3 AB	1	4.3	0.39	0.39
Granola	63.3	y	378.0	450.7	93.0	307.2 AB	1	1.2	0.30	0.31
Karlena	62.4	y	344.0	284.2	133.0	253.7 AB	1	11.9	0.30	0.31
Levante	59.4	y	96.7	281.0	501.0	292.9 AB	0	12.3	0.36	0.38
Lilly	67.7	y	399.8	528.3	57.0	328.4 AB	1	5.4	0.33	0.34
Lord	53.7	y	227.6	344.0	272.0	281.2 AB	1	2.3	0.34	0.30
Magnolia	54.9	y	187.2	264.8	119.0	190.3 AB	1	2.7	0.14	0.15
Mayan Gold	80.4	y	778.2	639.9	727.0	715.0 A	1	7.9	0.18	0.24
Michalina	53.7	y	205.0	534.8	113.0	284.3 AB	1	2.6	0.52	0.50
Noblesse	63.3	y	399.8	365.0	186.0	317.0 AB	1	8.2	0.28	0.31
Nofy	56.5	y	65.9	182.3	143.0	130.4 B	0	10.7	0.24	0.30
Omega	66.4	y	240.6	314.9	78.0	211.2 AB	1	10.0	0.44	0.37
Otolia	58.4	y	641.5	431.3	193.0	422.0 AB	0	2.6	0.36	0.32
Riviera	52.7	y	146.8	219.5	101.0	155.8 AB	1	2.6	0.55	0.50
Salome	63.2	y	596.3	407.1	658.0	553.8 AB	1	7.8	0.40	0.37
Sarpo Shona	50.3	y	182.3	245.4	104.0	177.3 AB	0	3.5	0.22	0.26
Slavnik	56.0	y	284.2	289.1	63.0	212.1 AB	1	3.3	0.40	0.27
Tajfun	54.9	y	546.1	476.6	20.0	347.6 AB	1	6.7	0.35	0.34
Tinca	58.5	y	171.8	237.3	85.0	164.7 AB	0	12.4	0.45	0.43
Triplo	64.4	y	323.0	329.5	96.0	249.5 AB	1	3.4	0.63	0.59
Twinner	65.3	y	260.0	392.5	1213.0	621.8 AB	1	14.8	0.53	0.51
Twister	59.7	y	465.3	298.8	126.0	296.7 AB	1	9.7	0.34	0.32
Voyager	55.4	y	395.0	363.4	60.0	272.8 AB	1	10.3	0.32	0.34
Wega	67.0	y	187.2	704.6	175.0	355.6 AB	0	7.7	0.31	0.34
Mean value	56.2		274.1	330.9	234.7	279.9	-	12.9	0.50	0.50

YI ^(1)^—yellow index, FC ^(2)^—flesh colour: y—yellow-fleshed; w—white-fleshed; TC ^(3)^—total carotenoid content; marker presence ^(4)^: 1—*Chy2* positive; 0—*Chy2* negative; VC ^(5)^—vitamin C; TAA ^(6)^—total antioxidant capacity measured by DPPH; TAA ^(7)^—total antioxidant capacity measured by FRAP; A, B, AB—homogenous groups.

**Table 2:** Sources of variation and one- and two-way ANOVA results for TC, VC, DPPH and FRAP in potato tubers.

Sources of Variation	ANOVA				
	Sum of Squares	Degrees of Freedom	Mean Square	F Statistic	Significance
	ANOVA results for TC				
Cultivar (C)	3.704.438	64	57.882	1.9978	***
Year (Y)	304.012	2	152.006	4.0722	**
(C) × (Y)	3.462.533	128	27.051		ns
	ANOVA results for VC				
Cultivar (C)	1285.732	64	20.090	2.4049	***
	ANOVA results for DPPH				
Cultivar (C)	1.48101	64	0.02314	2.496	***
	ANOVA results for FRAP				
Cultivar (C)	1.08066	64	0.01689	2.499	***

ns = not significant; ** significant at *p* < 0.01; *** significant at *p* < 0.001.

**Table 3:** Student’s *t*-test for the two independent groups of potato flesh colour (white and yellow) relative to TC, VC, DPPH and FRAP.

Variable	Student’s *t*-Test				
	Mean Yellow	Mean White	*t*	df	Significance
Total carotenoid (TC)	319.86 µg 100 g^−1^ DM	175.56 µg 100 g^−1^ DM	4.854	193	***
Vitamin C (VC)	6.49 mg 100 g^−1^ FM	5.84 mg 100 g^−1^ FM	0.670	63	ns
DPPH	0.38 µmol TE 100 mg ^−1^ DM	0.34 µmol TE 100 mg ^−1^ DM	1.276	63	ns
FRAP	0.38 µmol TE 100 mg ^−1^ DM	0.35 µmol TE 100 mg ^−1^ DM	1.010	63	ns

ns = not significant; *** significant at *p* < 0.001.

**Table 4:** Student’s *t*-test for the two independent groups of potato with or without the *Chy2* allele 3 marker relative to YI and TC.

Variable	Student’s *t*-Test				
	Mean *Chy2*+	Mean *Chy2*−	*t*	df	Significance
Yellow index (YI)	57.97	50.47	2.813	63	**
Total carotenoid (TC)	294.07 µg 100 g^−1^ DM	232.68 µg 100 g^−1^ DM	1.852	193	ns

ns = not significant; ** significant at *p* < 0.01.

## Data Availability

Supplementary data (climatic conditions, list of cultivars, results of chemical analyses) are available at https://doi.org/10.5281/zenodo.8012892.

## Supplementary Materials

The following supporting information can be downloaded at: https://www.mdpi.com/article/10.3390/ijms241411716/s1.
