# Supplementary material for: The Content of Total Carotenoids, Vitamin C and Antioxidant Properties of 65 Potato Cultivars Characterised under the European Project ECOBREED
Source: Int J Mol Sci. 2023 Jul 20;24(14):11716. doi: 10.3390/ijms241411716 (PMC10380281; doi:10.3390/ijms241411716)

<u>Supplementary Materials</u>

Table S1. Geographic, soil, fertilization, and fore crops data for field experiments.

Location	Geographic coordinates	Type of soil	Year	Pre-crop	Doses of fertilizers kg ha^{-1}
Młochów	52° 3′ 0″ N<br>20° 46′ 7″ E	Podzols	2019	rape	36 N, 30 P_2O5, 54 K_2O<br>25t/ha composted manure
			2020	oat	36 N, 30 P_2O_5, 54 K_2O<br>25t/ha composted manure
			2021	oat	34 N, 26 P_2O_5, 50 K_2O<br>25 t/ha composted manure

Figure S1. Rainfall (total monthly in mm) and temperature (average in monthly °C) during the A) 2019, B) 2020 and C) 2021 potato growing seasons in Młochów.

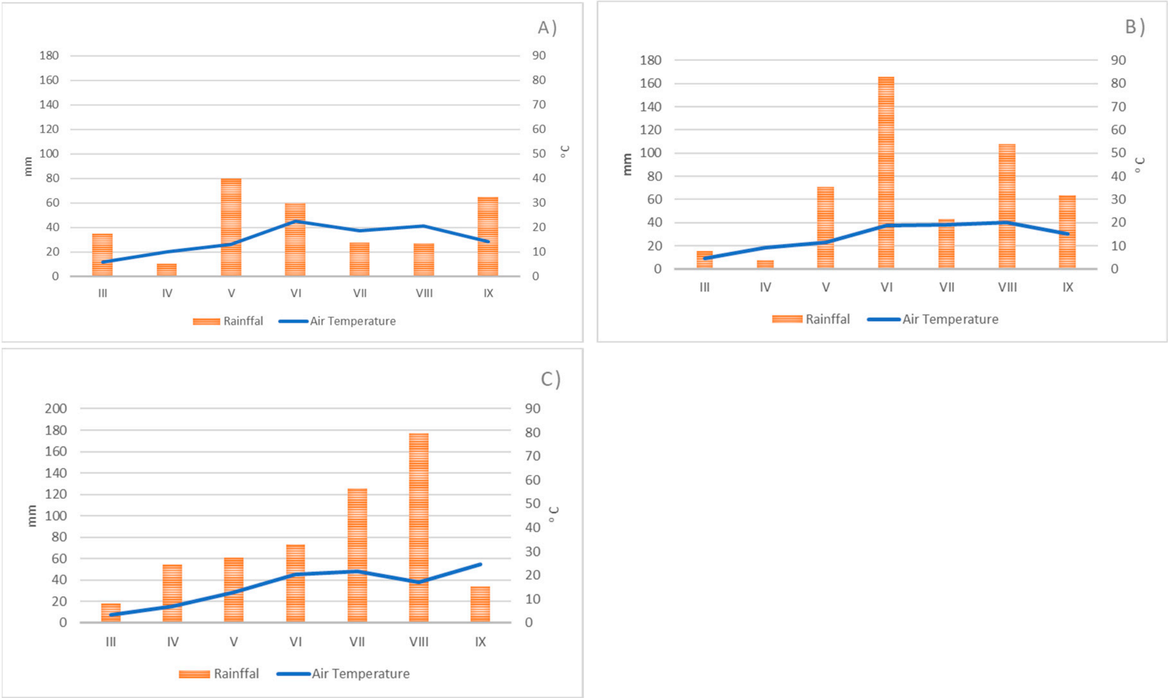

Supplement: Supplementary file 1 [file ijms-24-11716-s001.zip › ijms-2473902-supplementary.pdf]
